# “A Large Hiatal Hernia”: Atypical Presentation of Gastric Volvulus

**DOI:** 10.5811/cpcem.2017.2.31075

**Published:** 2017-06-06

**Authors:** Amirali Kiyani, Manraj Khosla, Veronika Anufreichik, Keng-Yu Chuang

**Affiliations:** * Maricopa Medical Center, Department of Internal Medicine, Phoenix, Arizona; † St. Joseph’s Medical Center, Department of Internal Medicine, Phoenix, Arizona; ‡ Creighton University School of Medicine, Omaha, Nebraska; § University of Arizona College of Medicine, Phoenix, Arizona

## Abstract

Gastric volvulus is a rare condition defined as an abnormal rotation of the stomach by more than 180 degrees. Gastric volvulus could present atypically with simply nausea and vomiting. A high index of suspicion is required for prompt diagnosis and treatment, especially when a patient presents with subacute intermittent gastric volvulus. Here, we present the case of a 56-year-old female with lung cancer status post left lower lobectomy undergoing chemotherapy who presented with intermittent nausea and upper abdominal pain for a few weeks. Barium study and computed tomography revealed acute mesenteroaxial gastric volvulus and she was treated with urgent surgical intervention.

## INTRODUCTION

Acute gastric volvulus is rare and is considered a medical emergency. Acute gastric volvulus usually presents with Borchadt’s triad: unproductive retching, epigastric pain and distention, and the inability to pass a nasogastric tube. Early recognition of the condition followed by surgical correction of the gastric malrotation can be life-saving. Prompt diagnosis of intermittent gastric volvulus may be challenging, as a patient might not exhibit classical symptoms in Borchadt’s triad and be mistaken with more common causes of intermittent abdominal pain such as peptic ulcer disease, dyspepsia or cholecystitis.

## CASE REPORT

A 56-year-old female with history of non-small cell lung cancer, hypertension, recently treated *H. pylori* infection and diabetes presented to a local emergency department (ED) twice in a week with the complaints of worsening nausea and bilious emesis over a one-month period while she was receiving chemotherapy. The patient also reported pain in the epigastrium that slowly progressed to the left chest. Patient had had left lower lobectomy with partial diaphragmatic resection a year prior as part of the treatment for the lung cancer. The patient had normal complete metabolic panel, magnesium, and lipase and a chest radiograph that was read as unremarkable besides “a large hiatal hernia.” She was discharged home the first time with antiemetics, but it was decided to admit her at the second ED visit as she had laboratory abnormalities significant for severe hypokalemia (potassium: 2.4 mg/dL) and hypomagnesemia (magnesium: 0.6 mg/dL).

**Image 1:**
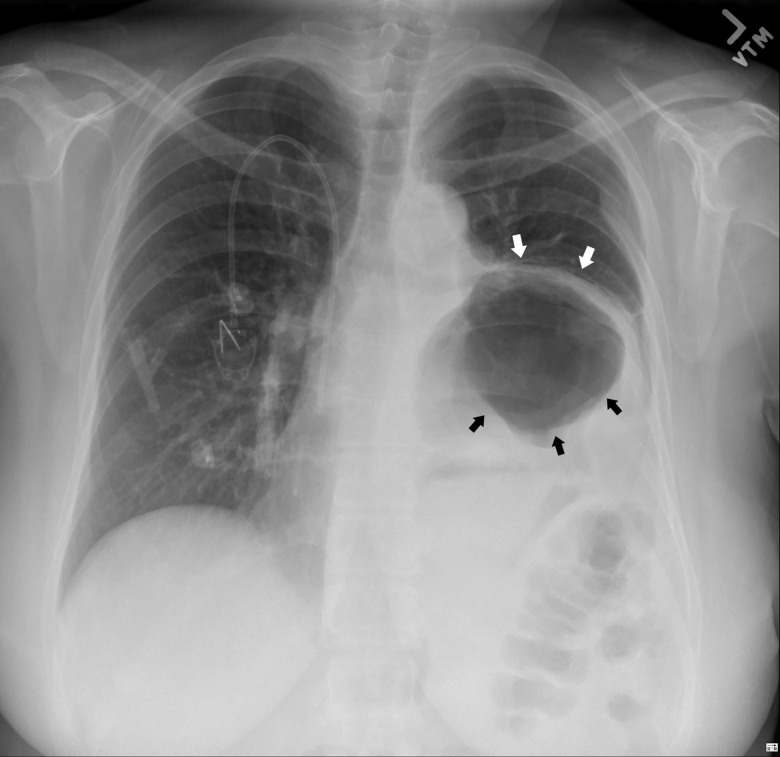
Chest radiograph showing an air-filled, thick-walled structure (black arrows) overlying the left lower thorax with marked elevation of the left hemi-diaphragm (white arrows).

**Image 2:**
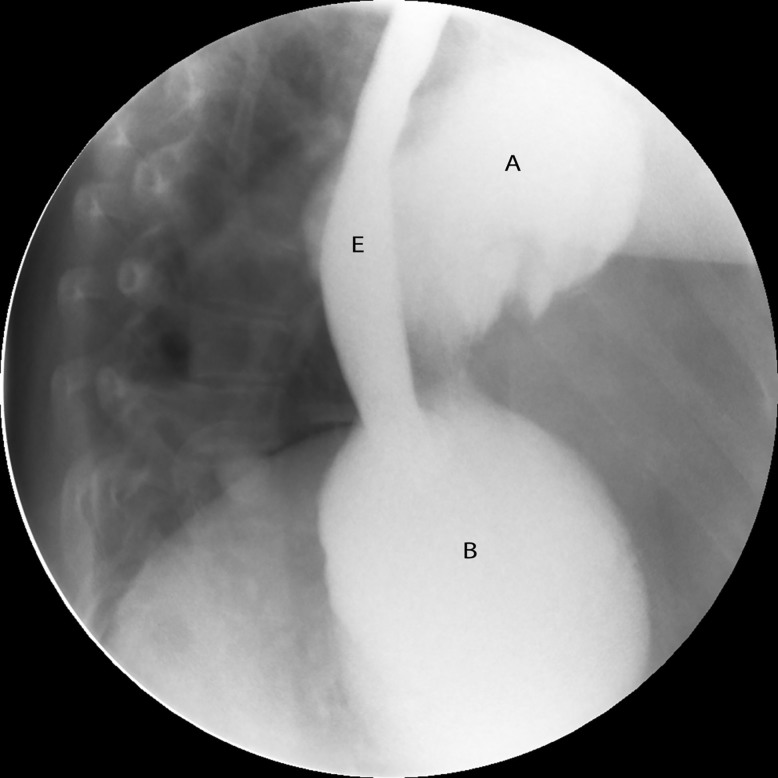
Barium UGI series demonstrating mesenteroaxial gastric volvulus. Antrum (A). Gastric body (B). Esophagus (E). *UGI,* upper gastrointestinal

**Image 3:**
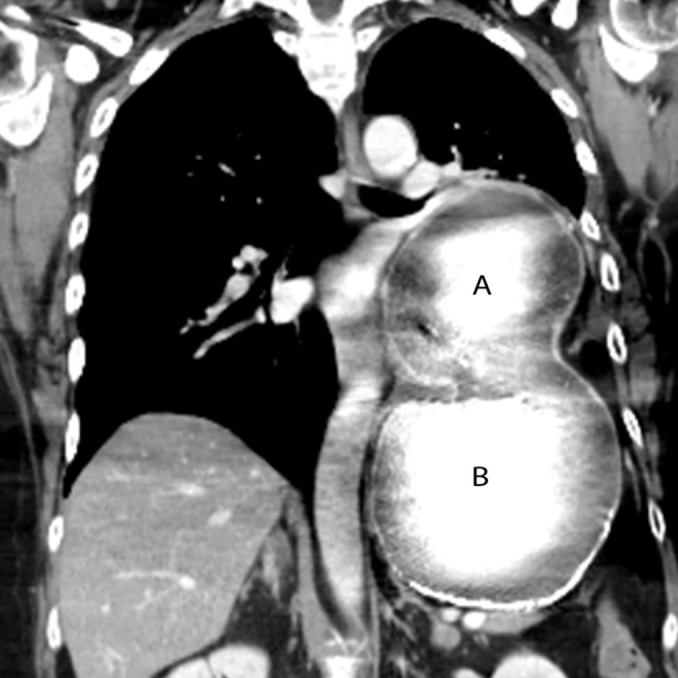
Mesenteroaxial volvulus was seen on a coronal CT of the chest and abdomen where the antrum (A) has migrated above the left diaphragm and is cephalad to the gastric body (B). *CT,* computed tomography

**Image 4:**
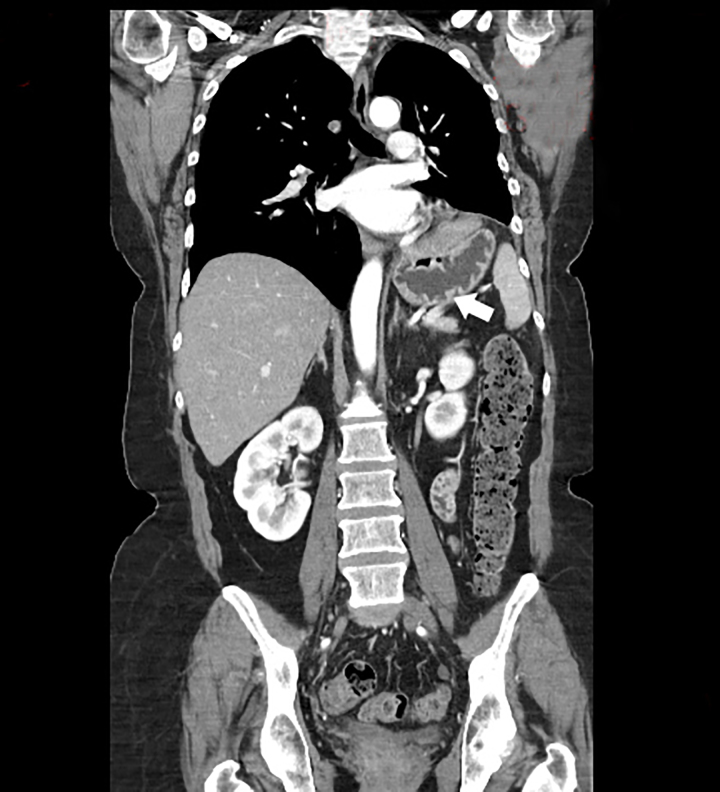
A control chest CT shows gastric body in normal anatomical position. *CT,* computed tomography

Physical examination was remarkable for decreased left lower lobe breath sounds as well as palpable tenderness in the epigastrium and the left chest wall. A chest radiograph revealed an air-filled, thick-walled structure overlying the left lower thorax with marked elevation of the left hemi-diaphragm (Image 1). The gastrointestinal (GI) service was requested to see the patient, and the consultant recommended a barium upper GI (UGI) series to define the gastric anatomy prior to performing an upper endoscopy. The barium UGI series revealed that about half of her stomach had migrated into the left thorax, occupying the space where her lung had been removed: the antrum was now situated above the diaphragm and was cephalad to the gastric body. Furthermore, the oral contrast administered could not empty into the duodenum despite the patient being placed in different positions (Image 2). A contrast-enhanced computed tomography (CT) of the chest, abdomen and pelvis next was performed and confirmed the diagnosis of mesenteroaxial gastric volvulus with gastric outlet obstruction (Image 3). The patient subsequently underwent emergent exploratory laparotomy for volvulus reduction, diaphragmatic defect repair and left tube thoracostomy. She tolerated the procedure well and experienced no complications. A control CT showed that the gastric body was in normal anatomic position (Image 4).

CPC-EM Capsule

What do we already know about this clinical entity?

Gastric volvulus is a rare but known entity. CT scan or upper GI series are the preferred diagnostic modalities. Treatment is surgical in most cases.

What makes this presentation of disease reportable?

Our patient presented with intermittent gastric volvulus, which can be very challenging to diagnose since it can mimic more common diseases such as peptic ulcer disease.

What is the major learning point?

Physicians should consider gastric volvulus as a rare but possible differential diagnosis in patients with compatible history and exam but uncommon presentation.

How might this improve emergency medicine practice?

Any delay in diagnosis of gastric volvulus can have grave consequence and emergency physicians should have a high index of suspicion for prompt diagnosis and treatment.

## DISCUSSION

Gastric volvulus is defined as an abnormal rotation of the stomach by more than 180 degrees and is classified as organoaxial (59%), mesenteroaxial (29%) or mixed.^1^ Mesenteroaxial volvulus is more likely found in the pediatric population and is rarely described in adult individuals.^2^ Risk factors for gastric volvulus include patient age over 50, gastric ligament laxity, pyloric stenosis, rectal atresia, gastroduodenal tumors, diaphragmatic injury and eventration, left lung resection, or pleural adhesions.^2^,^3^ Complications associated with gastric volvulus include bowel obstruction, strangulation, ischemia, necrosis, perforation and abdominal sepsis. Acute gastric volvulus is a medical emergency with mortality rates as high as 30–50%.^4^ The diagnosis of gastric volvulus mainly relies on barium UGI series. A CT of the abdomen can confirm the gastric malrotation and define the transition point.^5^,^6^ Upper abdominal defects including diaphragmatic eventration, paraesophageal hernia and wandering spleen can be seen associated with gastric volvulus on imaging studies. Gastric volvulus can sometimes be diagnosed through upper endoscopy and a tortuous appearance of the stomach; difficulty or inability for the endoscope to reach the pylorus can be encountered. Management is surgical and primarily involves decompression of the stomach, volvulus reduction and possible gastropexy or gastrostomy tube placement. Intra-abdominal defects should be corrected if contributory.^4^

Our patient with mesenteroaxial volvulus did not present with the typical Borchadt’s triad. Instead, she presented with migrating pain from the epigastrium to the left chest over a one-month period, likely due to intermittent volvulus corresponding to the process of the stomach migrating through the post-lobectomy diaphragmatic defect leading to the eventual acute volvulus. The diagnosis of gastric volvulus was not made during her first ED visit, as her symptoms, laboratory testing and the chest radiograph finding of “a large hiatal hernia” did not raise enough concern for additional imaging studies to be pursued. Fortunately, when she returned a week later, the correct diagnosis of gastric volvulus was made right away using barium UGI series and CT.

This experience demonstrates the importance of considering gastric volvulus as a rare but possible differential diagnosis when a patient has surgical history of left lobectomy and diaphragmatic injury, even if the patient does not present with a symptomology consistent with classic Borchadt’s triad. Lack of prompt diagnosis could gravely change the patient’s outcome.
